# Growth Hormone(s), Testosterone, Insulin-Like Growth Factors, and Cortisol: Roles and Integration for Cellular Development and Growth With Exercise

**DOI:** 10.3389/fendo.2020.00033

**Published:** 2020-02-25

**Authors:** William J. Kraemer, Nicholas A. Ratamess, Wesley C. Hymer, Bradley C. Nindl, Maren S. Fragala

**Affiliations:** ^1^Department of Human Sciences, The Ohio State University, Columbus, OH, United States; ^2^Department of Health and Exercise Science, The College of New Jersey, Ewing, NJ, United States; ^3^Biochemistry and Molecular Biology, The Pennsylvania State University, University Park, PA, United States; ^4^Department of Sports Medicine, School of Health and Rehabilitation Sciences, University of Pittsburgh, Pittsburgh, PA, United States; ^5^Quest Diagnostics, Secaucus, NJ, United States

**Keywords:** anabolic, catabolic, protein synthesis, skeletal muscle, endocrine, glucocorticoid, androgen, signaling

## Abstract

Hormones are largely responsible for the integrated communication of several physiological systems responsible for modulating cellular growth and development. Although the specific hormonal influence must be considered within the context of the entire endocrine system and its relationship with other physiological systems, three key hormones are considered the “anabolic giants” in cellular growth and repair: testosterone, the growth hormone superfamily, and the insulin-like growth factor (IGF) superfamily. In addition to these anabolic hormones, glucocorticoids, mainly cortisol must also be considered because of their profound opposing influence on human skeletal muscle anabolism in many instances. This review presents emerging research on: (1) Testosterone signaling pathways, responses, and adaptations to resistance training; (2) Growth hormone: presents new complexity with exercise stress; (3) Current perspectives on IGF-I and physiological adaptations and complexity these hormones as related to training; and (4) Glucocorticoid roles in integrated communication for anabolic/catabolic signaling. Specifically, the review describes (1) Testosterone as the primary anabolic hormone, with an anabolic influence largely dictated primarily by genomic and possible non-genomic signaling, satellite cell activation, interaction with other anabolic signaling pathways, upregulation or downregulation of the androgen receptor, and potential roles in co-activators and transcriptional activity; (2) Differential influences of growth hormones depending on the “type” of the hormone being assayed and the magnitude of the physiological stress; (3) The exquisite regulation of IGF-1 by a family of binding proteins (IGFBPs 1–6), which can either stimulate or inhibit biological action depending on binding; and (4) Circadian patterning and newly discovered variants of glucocorticoid isoforms largely dictating glucocorticoid sensitivity and catabolic, muscle sparing, or pathological influence. The downstream integrated anabolic and catabolic mechanisms of these hormones not only affect the ability of skeletal muscle to generate force; they also have implications for pharmaceutical treatments, aging, and prevalent chronic conditions such as metabolic syndrome, insulin resistance, and hypertension. Thus, advances in our understanding of hormones that impact anabolic: catabolic processes have relevance for athletes and the general population, alike.

## Introduction: the Complexity Of Hormone Signaling

**Figure 1:**
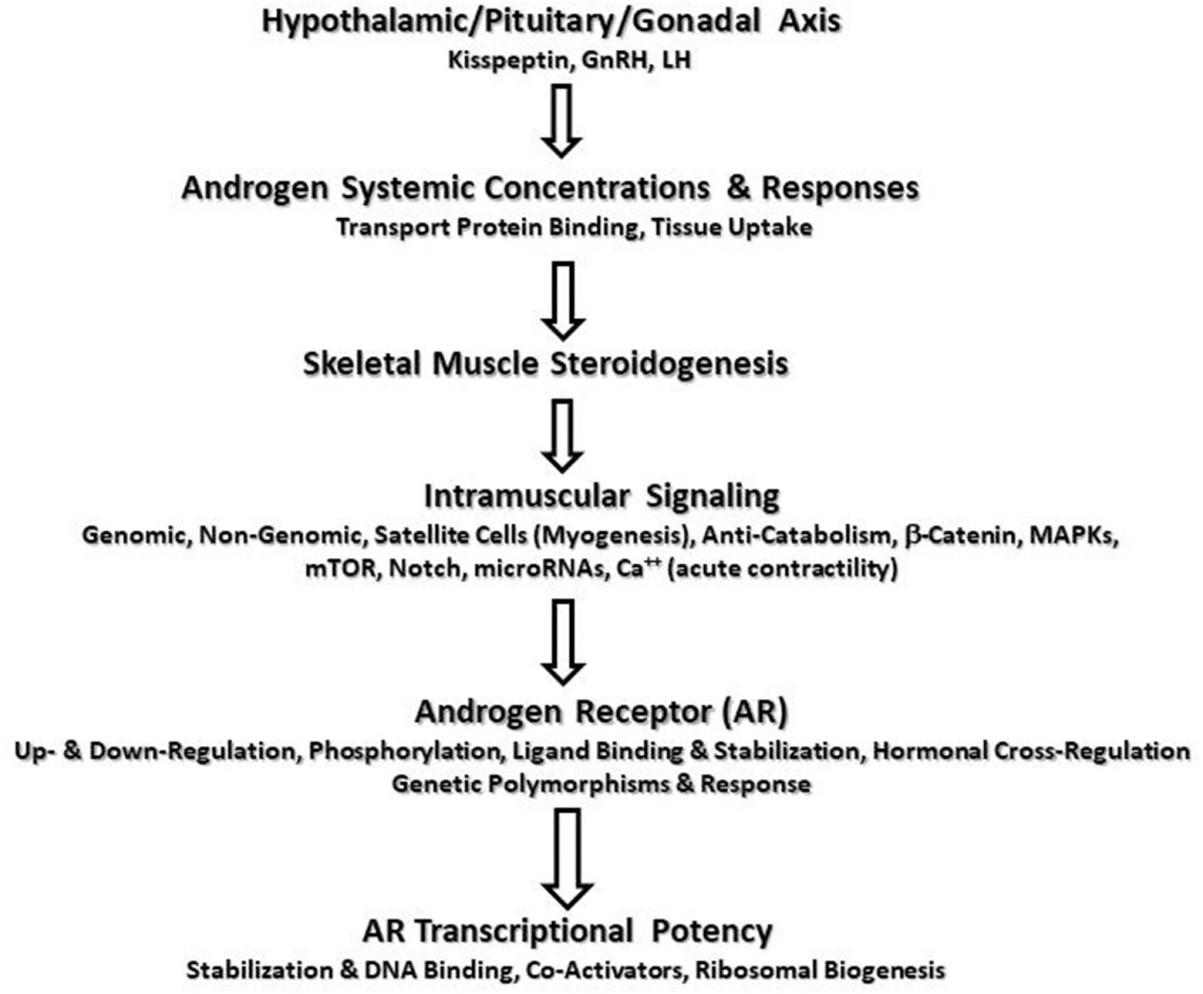
Potential sites of augmented androgen signaling responses or adaptations to resistance exercise.

Cell signaling may be described as a critical part of communication that governs basic activities of cells and coordinates all cellular actions. Hormonal signaling is part of a complex system involving a plethora of molecules. The actions and potency of any hormone will be affected by all components of the signaling chain (Figure 1). Depending on the cellular environment and negative feedback control, some components of the chain may be more proactive in eliciting a response or adaptation. To make a simple analogy, hormone signaling is analogous to playing a team sport. All players on the team have distinct roles and responsibilities during each play. Success depends on how well the team executes and communicates in an integrative manner to carry out team objectives. Hormones work in a similar manner. All stages from production, release, transportation, tissue uptake, and intracellular signaling must be considered in an integrative manner to accurately portray the effects of the hormone-receptor interaction (1). Thus, viewing only a fraction of the signaling chain may underrepresent the entirety of the hormonal actions. Science has shown the great complexity of hormonal signaling as strides have been made in cell biology and biochemistry.

## Testosterone Signaling Pathways, Responses, and Adaptations to Resistance Training

Testosterone (T) is an anabolic-androgenic steroid hormone that primarily interacts with androgen receptors (AR) in skeletal muscle whereas the more-potent dihydrotestosterone (DHT) primarily acts within sex-linked tissues with a possible secondary role in skeletal muscle (2) Although skeletal muscle content of DHT has been correlated to muscle strength and power (3), T replacement with and without dutasteride or finasteride (5α-reductase inhibitors) produces similar increases in lean tissue mass and muscle strength (4, 5). Thus, it is currently unclear if DHT is more anabolic in skeletal muscle than T alone. Testosterone performs a multitude of ergogenic, anabolic, and anti-catabolic functions in skeletal muscle and neuronal tissue leading to increased muscle strength, power, endurance, and hypertrophy in a dose-dependent manner (1). Genomic androgen/AR binding may alter the expression of more than 90 genes, several of which are involved in the regulation of skeletal muscle structure, fiber types, metabolism, and transcription (6). Studies show androgens increase protein synthesis rates, and reduce protein catabolism and autophagy (7). Castration reduces several markers of ribosome biogenesis that may only be partially restored by androgen treatment coupled with castration (8). In addition, evidence indicates that androgens may play a role in stimulating physical activity in males (9). Thus, androgens play important roles, in part, in mediating skeletal muscle protein synthesis and adaptations to resistance training (RT).

The primary androgen, T, is synthesized from cholesterol and other precursors in the Leydig cells of the testes (>95% in men with some adrenal contributions) under control of the hypothalamic-anterior pituitary-gonadal axis where gonadotropin releasing hormone (GnRH) stimulates the release of luteinizing hormone (LH) from gonadotrophs. GnRH functions under the control of hypothalamic neuropeptides such as kisspeptins, neurokinin-B, dynorphin-A, and phoenixins (10, 11). Kisspeptin (a 54 amino acid peptide) is encoded from the *KISS1* gene and is released from neurons within the arcuate nucleus and anteroventral periventricular nucleus of the hypothalamus as well as other tissues outside of the CNS. Kisspeptin binds to KISS1R (GPR54) receptors on GnRH neurons and causes the release of GnRH (via a G-protein 2nd messenger system). Hypothalamic neuropeptide expression is dependent on metabolic status (12); however, little is known regarding exercise responses. Khajehnasiri et al. (13) examined moderate vs. intense treadmill training for 6 months in rats and showed intense exercise (but not moderate) led to decreased GnRH mRNA and serum total T (TT) and LH. No differences were seen in kisspeptin mRNA although some differences were seen neurokinin-B and pro-dynorphin mRNA. Short-term administration of kisspeptin (Kp-54) or kisspeptin analogs (i.e., Kp-10) increase LH and TT in a dose-dependent manner in men with increases in LH but little change in TT in women (11, 14).

In women, ovarian and adrenal production of androgens are major sources (15). Skeletal muscle contains the enzymes and produces small amounts of androgens (16, 17). Testosterone is released into circulation and transported mostly by sex hormone-binding globulin (SHBG) (44–60%) and loosely-bound to albumin or other proteins. Free (unbound, up to 2% in circulation) T (FT) is taken up by tissues for binding to membrane-bound or cytoplasmic ARs and subsequent cellular signaling. However, some evidence suggests the possibility of an alternative mechanism to the “free hormone” hypothesis where membrane-bound receptor proteins (e.g., megalin—a low-density lipoprotein endocytic receptor) have been identified in multiple tissues including skeletal muscle, although the ability to internalize the bound steroid hormone complex and enable uptake via endocytosis still remains to be elucidated (18, 19). Nevertheless, SHBG concentrations influence T binding capacity and FT available for diffusion across the cell membrane. The presence of G-protein coupled receptors for SHBG in skeletal muscle membranes has been suggested to influence (i.e., inhibit or stimulate) non-genomic androgen signaling via modulation of adenylate-cyclase with cAMP synthesis and activation of protein kinase A (20), although it is currently unclear as to the magnitude of, if any, impact it may have during androgen signaling. Some T is converted to the more potent DHT via 5α reductase. This enzyme is present in skeletal muscle and circulating DHT can diffuse into muscle cells and bind to ARs with higher affinity than T. Some T is aromatized to estrogens, and final metabolism of T occurs in the liver and kidneys where inactivated metabolites are excreted in urine.

The responses of T to RT in men and women have been extensively reviewed (2, 21). Most studies show significant elevations of TT and FT in men through 30 min into recovery with few changes in resting baseline concentrations. In women, studies show no or limited acute elevations. The magnitude of the acute responses is affected by many factors including the demands of the protocol, nutritional intake, training experience but mostly due to plasma volume reductions and reduced clearance (1). The ramifications of acute elevations during RT are unclear but appear to be part of the macro-signaling cascade affecting, in part, muscular adaptations. Some studies indicate relationships between T elevation and AR up-regulation, strength and hypertrophy enhancement (22–25) whereas other reports indicated no such relationships (26). These conflicting results demonstrate the complexity of hormonal responses and the likelihood several factors are contributing to the response. Acute T responses must be viewed within the context of multiple skeletal muscle signaling pathway adaptations as well the well-known interaction between T signaling and other hormone signaling pathways involving the GH isoforms and aggregates, IGF-I and mechano-growth factor (MGF), insulin, and cortisol responses (27–29).

## Skeletal Muscle Steroidogenesis

Skeletal muscle steroidogenesis from DHEA is another potential source of T (16). Steroidogenic enzyme content and T concentrations in skeletal muscle are similar between men and women (17). In older men, 12 weeks of RT increases skeletal muscle DHEA, FT, DHT, 3β-hydroxysteroid dehydrogenase (3β-HSD), 17β-hydroxysteroid dehydrogenase (17β-HSD), 5α-reductase type I content, and AR protein content (30). The increased DHT and FT were related to increased isokinetic strength, muscle CSA, and power (30). However, RT studies in younger men and women show no changes in muscle T or steroidogenic enzymes (17, 31). However, responders to RT were shown to increase 5α-reductase (31). It is possible that increased muscle steroidogenesis may be a mechanism to help counteract T reductions in older men undergoing RT but less likely in healthy, young men.

## Androgen Signaling Pathways

Historically, androgen signaling was thought to be governed predominately by classical genomic signaling common to steroids and steroid receptors. FT or DHT (or other synthetic anabolic steroid) binds to a cytoplasmic AR, dissociates from heat shock proteins, and the complex translocates to the nucleus to bind to specific androgen response elements on DNA. The androgen/AR complex serves as a transcription factor leading to protein synthesis with the help of co-activator proteins. Prior to androgen stimulation of skeletal muscle tissue, higher order muscle tissue activation is needed. Increased muscle strength, power, endurance, and hypertrophy resulting from RT begins with neural stimulation and the optimal recruitment of motor units based on the size principle. Androgen signaling increases neural transmission, neurotransmitter release, motoneuron cell body and dendrite size, and regrowth of damaged peripheral nerves (32). Androgen signaling in cerebral neurons is needed to maintain muscle mass in fast-twitch muscles despite elevations in circulating T (33). This may be regulated by reduced spontaneous locomotor activity. Thus, the RT stimulus is critical to activation of muscle tissue and the role of androgens in enhanced neural drive warrants further study. Genomic signaling accounts for a large magnitude of androgen actions; however, a number of other signaling pathways have been identified demonstrating the complexity of androgen signaling its impact on skeletal muscle development.

Non-genomic AR signaling has been a topic of interest and debate in recent times. Non-genomic actions are rapid with short latency periods acting independently (mostly at the cell membrane and cytoplasmic levels) of nuclear receptors (20). Non-genomic effects are thought to be mediated by direct binding to a target molecule, through intracellular AR activation (i.e., Src kinase), through a transmembrane AR receptor, or via changes in membrane fluidity (20). Non-genomic signaling may involve G-protein 2nd messenger system signaling. Non-genomic signaling may increase intracellular calcium concentrations (possibly affecting contractile properties) (34), stimulate activation of MAPK signaling (35), and mammalian target of rapamycin (mTOR) pathway signaling (36). Binding of bound or unbound T to ARs activate G-protein-linked receptor that activates PI3K and phospholipase C, increases IP_3_ which binds to receptors on the sarcoplasmic reticulum to liberate calcium. Calcium may activate Ras/ERK1/2 pathway signaling (34). Castration reduces Akt/mTORC1 signaling and AR protein expression whereas nandrolone decanoate administration has the opposite effect (37). Basulato-Alarcon et al. (36) showed T increased MTORC1/S6K1 pathway signaling possibly through PI3K activation of Akt. Zeng et al. (38) reported DHT implantation plus exercise in rats for 10 days increased AR and IGF-I mRNA and AR phosphorylation (Ser210). They reported greater muscle hypertrophy via mTOR signaling and suggested cross-talk between IGF-I signaling and non-genomic AR signaling was critical to the augmented combined effects. Non-genomic signaling occurs rapidly within seconds to minutes, much faster than classic genomic signaling which takes hours, and requires constant presence of androgens to maintain intracellular signaling. It is unclear if the increased intracellular calcium enhances force production (35). The impact of non-genomic signaling to training-related adaptations remains unclear; however, it appears the interaction between genomic and non-genomic signaling pathways appear critical to maximizing muscle hypertrophy (36). MAPK signaling may phosphorylate the AR increasing its transcriptional capacity.

Testosterone may be anti-catabolic by either decreasing glucocorticoid receptor (GR) expression, interfering with cortisol binding, or the AR-T complex may compete with cortisol-GR complex for *Cis*-element binding sites on DNA (and vice versa). DNA binding domains and ligand binding domains between the AR and GR are 79 and 50% homologous. Glucocorticoids increase expression of atrophy-related genes (i.e., atrogin-1, MuRF1, and forkhead box 01) and androgens reduce atrogene expression, reduce GC-related IGF-I expression inhibition, and down-regulate GR expression in skeletal muscle and muscle satellite cells (39).

Androgens mediate some anabolic functions through myogenesis via multiple pathways. Satellite cells and myoblasts possess ARs and androgen binding increases satellite cell activation, proliferation, mobilization, and differentiation, and incorporation into skeletal muscle (40). Androgens increase myogenesis via increased Notch signaling of satellite cells possibly due to reduced myostatin and increased Akt activation (41) and through increased expression of IGF-I in satellite cells and muscle fibers (28). Androgen binding to ARs on mesenchymal pluripotent cells increases their commitment to myogenesis rather than adipogenesis (42). Androgens may increase follistatin expression and decrease or increase myostatin and down-regulate or up-regulate its gene expression, down-regulate Acvr2b receptor mRNA and Smad 2/3 phosphorylation, decrease myostatin signaling molecules, increase myogenic marker up-regulation, e.g., MyoD, myogenin, myotube formation, and myonuclei number and central positioning (39, 42, 43).

Genomic androgen/AR binding may enhance muscle performance via stimulating the Wnt-β-catenin pathway. Wnt binds to frizzled/lipoprotein receptor protein 6 receptors and activates disheveled and inhibits glycogen synthase kinase-3 (GSK-3) reducing β-catenin dephosphorylation and increases its activity. The FT-AR complex inhibits GSK-3 and increases β-catenin where it translocates to the nucleus, binds to DNA response elements (T-cell factor/lymphoid enhancer factor 1 –TCF/LEF), increases transcription, and activation of muscle satellite cells. As β-catenin lacks a nuclear localization sequence and needs cytosolic proteins with a sequence to assist in translocation, androgen/AR complex may chaperone β-catenin to the nucleus where it binds to specific DNA elements. The presence of T increases AR-β-catenin interaction and transcriptional capacity. Positive correlations were shown between AR protein content and Wnt5 expression and muscle mass and Wnt5 expression in rats (44). Testosterone treatment increased Wnt5 protein expression and muscle size in a dose-dependent manner (44). Spillane et al. (45) reported significant up-regulation of VL muscle β-catenin following upper and lower body RT at 3 and 24 h PE and increased AR-DNA binding capacity and suggested the increased binding capacity was linked to greater β-catenin pathway signaling.

## The Importance of Androgen Signaling for Muscle Strength and Hypertrophy

Human and animal studies (using a variety of research models) demonstrated the importance of androgens for maintaining and increasing skeletal muscle strength and mass. Muscle strength and mass are reduced significantly (by up to 20%) in male AR knockout mice (6). In satellite cell-specific AR knockout mice, type II to I fiber conversions and reduced muscle strength have been shown (2014). Other muscle-specific AR knockout mice models have shown reduced lean tissue mass and fast-to-slow fiber type conversion without concomitant changes in muscle strength (46). Inoue et al. (47) showed that administration of an AR antagonist in rats (oxendolone) during 2 weeks of electrical stimulation of the gastrocnemius muscle attenuated 70% of stimulation-induced hypertrophy compared to the control condition. The same research group showed that electrical stimulation of rat gastrocnemius increased AR number by 25% within 3 days and the AR up-regulation preceded muscle hypertrophy. Deschenes et al. (48) showed RT in rats increased AR binding capacity in fast-twitch muscles (i.e., extensor digitorum longus) of rats but not slow-twitch (i.e., soleus). In humans, hypogonadism, aging, glucocorticoid use, obesity, and androgen deprivation therapy (ADT) are negative regulators of androgen actions. A study from Kvorning et al. (49) showed that 8 weeks of RT with or without the GnRH analog goserelin (that reduced TT to ≤2 nmol/L) significantly attenuated increases in isometric strength and leg lean tissue mass. The authors concluded that suppression of T below 10% of normal levels strongly attenuates the increase in lean tissue mass and muscle strength seen during RT (49).

## Androgen Receptor Physiology

The signaling effects of androgens are mediated through the AR which belongs to a family of steroid receptors. The AR gene is located on the q arm of chromosome X at position 11–12 and contains 8 exons that code for approximately 2,757 base pair open reading frames (50, 51). The first exon codes for the N-terminus transcription activation domain; exons 2–3 code for the central DNA binding domain; exons 4–8 code for the C terminus ligand-binding domain (50). The AR consists of ~920 amino acids (~110 kD or more when phosphorylated; and consists of 12 α-helices and 2 β-sheets) and is found in nearly all tissues in the human body and other truncated versions with biological activity have been identified (52). The presence of ARs correlates highly with the functions of androgens. AR activity may be altered by phosphorylation at several serine (and threonine and tyrosine) residues particularly in the transcription region (e.g., Ser81, 94, 213, 515, 651), and through methylation, ubiquitination, sumoylation, and acetylation at various sites (>23 sites). For example, phosphorylation of serine residue 651 is needed for full transcriptional activity (53). Phosphorylation may occur during ligand binding and through other signaling pathways indicating that the AR is cross-regulated by other ligand-receptor interactions (54). Thus, phosphorylation may augment androgen/AR transcriptional action (in the presence or absence of androgens) and demonstrate the high intracellular regulatory potential of the AR (55). The AR is activated through ligand-independent mechanisms including IGF-I induced MAPK-ERK1/2, p38, and JNK phosphorylation in C_2_C_12_ muscle cells (56). The AR may modulate its phosphorylation state to sensitize itself to anabolic signals in the presence of lower androgens. A recent study from Nicoll et al. (57) showed that men have higher baseline AR protein content than women; however, women had greater AR phosphorylation at rest at ser515 and ser81 residues indicating that the AR activity could be augmented independent of ligand levels.

## Androgen Receptor Binding, Stabilization, and Transcription

Ligand binding occurs at the C terminus of the AR. Upon androgen binding to the ligand binding domain (LBD), dissociation from the heat-shock proteins occurs, hyperphosphorylation, dimerization, and conformational changes occur converting the AR to a transcription factor that interacts with *androgen response elements or AREs* of DNA (58). The AR DNA binding domain contains zinc finger motifs that recognize both consensus and selective AREs. Androgen binding activates and stabilizes the AR and induces N → C terminus interaction which is selectively induced by high-affinity T and DHT, and lower-affinity anabolic steroids (e.g., oxandrolone, fluoxymesterone) (59). Greater stabilization is seen with DHT more so than T as T dissociates from the AR 3 times faster than DHT (60). Testosterone is the primary androgen interacting with ARs in skeletal muscle. Androgens have different potencies, in part, due to affinity and binding properties of the AR.

The androgen/AR complex serves as a transcription factor leading to increased protein synthesis. The N-terminal domain is responsible for transcription activation. Androgen binding to the AR completes the groove that serves as a recruiting surface for co-activators (attract co-regulator motifs, e.g., LxxLL, FxxLF) that form a bridge between the DNA-bound AR and the transcriptional machinery. Co-regulator proteins mostly interact with the N-terminus domain (with some binding at the LBD). More than 250 co-regulators exist many of which are co-activators (61). Co-activators augment transcriptional activity and enhance signaling, e.g., SRC-1, SRC-3, TIF-2, ARA24, ARA160, BAF57, BAF60A, ARA54, ARA70 whereas co-repressors (e.g., SMRT, SIRT1, Ankrd1) reduce transcriptional activity. Many co-activators involve chromatin remodeling, histone acetyltransferase, methyltansferse, and demethylase, DNA stabilization, and pre-initiation complex (PIC) recruitment whereas some corepressors tighten nucleosomes limiting accessibility (61). Micro RNAs have been shown to mediate AR function via co-repressor expression inhibition (62). The AR LBD coactivator binding groove is a target of drugs to manipulate AR activity especially in the development of anti-prostate cancer drugs (63). However, little is known regarding RT and potential up-regulation of co-activators which may serve as a great area of interest for future research.

Several models have been proposed to explain mechanism(s) involved in gene transcription including chromatin remodeling, direct binding of AR to proteins in the PIC such as transcription factors TFIIB (i.e., transcription factor IIB) and TFIIF (i.e., transcription factor IIF), and AR interactions with complexing proteins and/or co-regulators to enhance assembly of the PIC (64, 65). It appears that a multiple-step model that incorporates combinations of these models may be most accurate. Upon DNA binding and co-activator recruitment, the co-activators covalently modify targeted histone N termini to loosen the nucleosomal structure (via modifying the net charge) to facilitate transcription in the repressed chromatin (61). Transcriptional activation by AR ultimately requires the recruitment of RNA polymerase II to the promoter of target genes. The co-regulators, as well as the ligand-bound activation of AR, enhance assembly of and stabilize the *PIC*, which is the assembly of general transcription factors (64). Polymerase II recruitment is mediated through the assembly of the PIC, the first step of which is binding of TATA binding protein (TBP) near the transcriptional start site. TBP is part of a multi-protein binding complex with TFIID that induce bending of DNA, which brings the sequence of the TATA element closer to interact with general transcription factors and co-regulators. TFIIB binds directly to TBP and functions to recruit the TFIIF-polymerase II complex. TFIIF domains also serve in transcription initiation and elongation. ATPase, the kinase TFIIE, and helicase TFIIH are then recruited to polymerase II to facilitate DNA strand separation before transcription initiation.

## Androgen Receptor Polymorphisms and Performance

The first exon contains several regions of repetitive DNA sequences one of which is the CAG (polyglutamine) triplet repeat that begins at codon 58 and extends for >21 repeats. This length varies between 8 and 35 repeats (18–24 is most common). Another is a polyglycine (GGN) repeat in the transactivation region. Genetic polymorphisms yielding a variety of repeats are associated with a variety of conditions including male infertility, hypogonadism, prostate, and testicular cancer, bone disease, neurodegenerative, and cardiovascular disease (66). These could contribute, in part, to responder or non-responder status when examining training adaptations. Long CAG repeats may interfere with androgen actions whereas short repeats (CAG and GGN) are associated with increased AR protein expression and androgen action. However, contradictory results were shown where CAG repeat number was positively related, inversely related, or not related to lean body mass (LBM), T, or FT concentrations, and muscle strength in young and older men (67–70). Nielsen et al. (71) showed inverse relationships between CAG repeat number and thigh and trunk muscle size to where every reduction in repeats of 10 equaled an increase of muscle size by 4%. Thus, performance phenotypes based on AR candidate gene polymorphisms remain unclear.

## Androgen Receptor Up-regulation and Adaptations to Resistance Training

AR protein content is a critical variable in RT-induced androgen-mediated skeletal muscle protein accretion in healthy men (31). The concentration of ARs in skeletal muscle depends on the muscle fiber type, sex, training status, and androgen concentrations. Several studies investigated AR responses to RT (Table 1). Most studies show baseline AR protein content does not change although one study found significant down-regulation (85) and one study reported up-regulation in older men (30). The most expected pattern of change is acute up-regulation of AR mRNA and protein content within 1–2 days of RT followed by a return to baseline unless another workout is performed. Initially, AR protein content may not change or be down-regulated within the first 2 h PE in the fasted state (73). Post-workout protein/CHO feeding may ameliorate this response (77). Notable up-regulation of AR mRNA and protein is seen ~28 h PE (89) while is most pronounced 48 h PE (74, 75). The response is similar in young and old men (80) and may lessen over time with training experience (81). The AR mRNA and protein up-regulation correlated to TT and FT concentrations in the blood (19, 79). AR protein content explains a large amount of variance in muscle hypertrophy seen during RT (84), and its role may be potentiated with interaction of other hormones such as growth hormone and IGF-I.

**Table 1:** Summary of androgen receptor changes following resistance training.

**References**	**Subjects**	**Muscle**	**Protocol**	**Biopsy time**	**Results**
Kadi et al. (72)	UT men, PL on AAS, PL—no AAS	VL, TR	Cross-sectional comparison	BL	PL > % AR-positive myonuclei in TR than UT − P(AAS) > % AR-positive myonuclei than drug-free PL −↔ in VL between groups
Ratamess et al. (73)	RT men—fasted	VL	SQ: 1 or 6 sets of 10 reps, 80-85% 1RM−2-min RI	1 h PE	1 set = no change AR protein 6 sets = sig. ↓ AR protein − BL AR content correlated with 1RM squat strength
Bamman et al. (74)	UT men and women	VL	SQ: 8 × 8 ECC reps (~110% 1 RM) or CON reps (~85% 1 RM)	48 h PE	AR mRNA ↑ by 102% (CON) and 63% (ECC)
Willoughby and Taylor (75)	UT men	VL	SQ, LP, KE−3 sets of 8–10 reps each −75 to 80% 1RM, 3 min RI − 3 sessions separated by 48 h	48 h PE	AR mRNA ↑ 35, 68, 43% after each workout AR protein ↑ 40, 100, 202% after each workout − AR mRNA/protein correlated with PE TT and FT
Vingren et al. (76)	RT men and women—fasted	VL	SQ: 6 × 10 reps −80% 1RM, 2-min RI	10 and 70 min PE	AR protein ↓ at 10 min in women; ↓ at 70 min in men and women − AR protein men > women
Kraemer et al. (77)	RT men	VL	SQ, BP, BOR, SP: 4 × 10 reps each 80% 1RM, 2-min RI − Water + L-carnitine or feeding + L-carnitine post RE	1 h PE	Feeding ↑ AR protein
Spiering et al. (25)	UT men—fasted	VL	5 × 5RM KE following rest (CON) or after upper body RE eliciting TT ↑ by 16% (HT)	10 and 180 min PE	AR protein at 180 min tended to ↓ in CON, ↔ in HT from REST; AR protein following HT > CON
West et al. (78)	MT men and women—fed PE	VL	LP−5 × 10 reps; leg ext/curl superset 3 × 12 reps, 1 min RI	1, 5, 26, 28 h PE	↔ AR mRNA at 1, 5 h AR mRNA ↑ 28 h > 26 h
Poole et al. (19)	UT young and older (60–75 years) men	VL	9 sets of lower-body RE, 10 reps each set, 80% of 1RM, 2-3 min RI—completed 3 workouts	24, 48 h PE	↔ AR mRNA 48 or 24 h post RE over 3 days AR mRNA young men > old − PE TT at 30 min correlated to AR mRNA
Hulmi et al. (79)	RT older (57–72 years) men	VL	Whey or placebo: LP−5 × 10RM, 2-min RI	1 and 48 h PE	AR mRNA trend for ↑ in whey group; when groups combined sig. ↑ in AR mRNA 1 and 48 h Trend for ↑ AR protein in placebo 1 h − Change in AR mRNA 1 h correlated to PE TT response
Ahtiainen et al. (80)	UT young and older (60–65 years) men	VL	Acute RE before & after 21 weeks of RT: protocol—LP – 5 × 10RM, 2 min RI	1 and 48 h PE	↔ Acute AR protein and mRNA response over 21 weeks − AR response correlated to 1RM strength, LBM, and CSA −↔ BL AR mRNA/protein between old & young men or after RT
Ahtiainen et al. (81)	RT young men	VL	LP – 5 × 10RM, SQ – 4 × 10RM, 2 min RI	1 and 48 h PE	↔ AR mRNA and protein
Ahtiainen et al. (82)	UT young and older (70–75 years) men	VL	Acute RE before and after 12 months of lower-body RT: protocol—LP – 5 × 10RM, 2-min RI	IP (0) and 2 h PE	↔ AR content 0 and 2 h Chronic: ↔ BL VL AR content − No difference between BL VL AR content in young and old men
Kvorning et al. (27)	Young men, limited RT experience	VL	8 weeks of RT: GnRH analog (goserelin, 3.6 mg 3 times to ↓ TT) or placebo; acute RE pre and post RT	BL, 4, 24 h PE	Blocked TT and RT had no effect on AR mRNA acute or chronic at BL
Nilsen et al. (83)	Men with prostate cancer on ADT	VL	16 weeks of RT	BL	↔ BL AR protein content
Sato et al. (30)	UT young and older (mean = 67 years) men	VL	12 weeks of RT: 3 days/week, KE and LC−3 × 10 reps, 70% of 1RM, 3-min RI	BL	AR protein in young men > old men − BL AR protein ↑ in old men (no post biopsies taken for young men)
Morton et al. (31)	Young RT men	VL	12 weeks of RT: high reps (20–25 reps with 30–50% of 1RM) or low reps (8–12 reps with 75–90% of 1RM)	BL	↔BL AR protein content over 12 weeks − AR protein content in responders > non-responders
			− Divided subjects into responders vs. non-responders		− AR protein content correlated with LBM, type I, and type II muscle CSA
Mitchell et al. (84)	UT young men	VL	16 weeks of RT: 4x/wk—upper/lower body split: 3 × 6–12 reps, 1–2 min RI	BL	↔ BL AR protein content −Δ AR protein correlated to fiber CSA − AR protein & p70S6K phosphorylation accounted for 46% of variance in size
Mobley et al. (85)	UT young men	VL	12 weeks of RT: 3 days/week, 5 exercises —undulating periodization, 4–10 reps	BL	↓ BL AR protein content similar in low, moderate, and high responders
Haun et al. (86)	Young previously RT men	VL	6 weeks of RT: 3 days/week, 10 reps per set, 60% of 1RM, 10–32 sets per exercise per week	BL	↔ BL AR protein content in high and low responders
Roberts et al. (87)	UT young and older (mean = 68 years) men	VL	Acute RE: SQ, LP, KE −3 × 10 reps, 80% of 1RM, 3 min RI	24 h PE	BL AR mRNA in older men > young −↔ AR mRNA 24 hrs PE in either group − FT negatively correlated with AR mRNA
Brook et al. (88)	UT young and older (~69 yrs) men	VL	6 weeks of RT: unilateral KE, 6 × 8 reps 75% of 1RM	BL, 90 min PE	↔ BL AR mRNA ↔ PE AR mRNA

*PL, powerlifters; AAS, anabolic-androgenic steroids; BL, baseline; IP, immediate post exercise; RT, resistance trained; RE, resistance exercise; 1RM, one repetition-maximum; RI, rest intervals; VL, vastus lateralis; TR, trapezius; PE, post exercise; UT, untrained; MT, moderately trained; ECC, eccentric; CON, concentric; SQ, squat; LP, leg press; KE, knee extension; LC, leg curl; BP, bench press; BOR, bent-over row; SP, shoulder press; TT, total testosterone; FT, free testosterone; LBM, lean body mass; CSA, cross-sectional area; ↑ increase; ↓ decrease; ↔ no change*.

## Growth Hormone: A New Complexity With Exercise Stress

The concept that a “hormone” caused growth was first proposed in 1911 (90). Since that time, and as noted on PubMed, >126,000 publications have reported on some feature of growth hormone (GH). Of that large number, comparatively few (~2,800) address its role in human exercise. In turn, only a small subset of these exercise studies considered the issue and importance of GH assay choice employed and the large difference it can make in interpreting experimental data. The purpose of this review is to (a) briefly review early history of GH bioassays, (b) summarize the data base that addresses the relevance of assay choice in performing exercise stress studies in humans, and (c) suggest how emerging data concerning GH processing in the pituitary gland may offer new direction(s) for the study of this anabolic hormone in health and aging.

## Early History of GH Bioassay

The isolation of GH from pituitary extracts of many mammalian species, using biochemical techniques available at that time [~1950's−1970's], was described in a review by Papkoff and Li (91). During this early period, the three most often used growth bioassays were (a) the weight gain assay in the plateaued female rat; (b) the weight gain assay in the immature hypophysectomized rat; and (c) the tibia test; an assay originally proposed by Greenspan et al. (92) that measured bone growth at the tibial plate of the hypophysectomized rat following a 4 day injection of GH test sample. In addition, investigators also used other types of biological assays to measure circulating GH hormone that had other endpoints (e.g., lipolysis, carbohydrate metabolism). In fact, results from such differing assay approaches led C.H. Li to propose that a better name for the hormone might be “metabolic hormone” (93). To the best of our knowledge, it was also during this time period (1965) that the first study documenting that human exercise was a potent stimulus for the release of GH from the pituitary appeared (94).

A 15 year period (~1970–1985) marks the time when a majority of clinical and basic investigators appear to have transitioned from measuring circulating GH by biological assay to immunoassay. During this transition period, a critically important experimental series by Ellis et al. (95) was designed to compare results generated between rat growth assays and GH immunoassays. Their data unequivocally showed that bioassays and immunological assay results did not correlate. Plasma GH concentrations measured by this *in vivo* bioassay were estimated to be much greater (~300x) than those measured by immunoassay. Further, in 1978 this group reported that a pituitary growth factor, which escaped detection by immunoassay, nevertheless had strong GH activity in the established rat tibia bioassay (95). Their biochemical studies indicated that this factor was relatively large (~80 kDa). Moreover, the relative concentrations of bioactive GH in the rat pituitary and/or circulation (including human plasma) changed differentially in response to a variety of physiological stimuli (e.g., cold stress, fasting, insulin injection). This study was largely ignored. In retrospect, the authors of this review believe that this pioneering study should have had a more significant impact on future GH research efforts than it did. The ramifications of this concept for the multi-dimensionality of the many GH isoforms are further delineated in a recent review (96).

## GH Isoforms

A comprehensive review of GH variants, their isolation, availability, and physiological activities is beyond the scope of this review. However, the following points help establish the thesis of this review, viz. that other potent hGH bioactive forms are present in the pituitary and plasma. However, many remain to be fully characterized, both physiologically and structurally. It is clear: GH is not a single substance.

After gene cloning, the first recombinant human GH (rhGH) was produced biosynthetically in 1979 by Genentech (San Francisco, California). Work on this product showed that the 191 amino acid isoform (22 kDa) was identical to a native molecule present in the pituitary gland and plasma (97). This form was active in the tibial bioassay as well as other bioassays having the growth endpoint. Two factors; viz. (a) availability of the recombinant product and (b) closure of the National Pituitary Agency (in 1985) for production of hGH extracted from human pituitary glands, led to overwhelming use of antibody- based technology (e.g., polyclonal, monoclonal antibodies) and less frequently used cell- based bioassays for GH measurements.

In the ~30 years following the Ellis report, pioneering biochemical experiments from many laboratories (Lewis, Sinha, Kostyo, and Baumann to name but a few) led to the now familiar realization, summarized by Baumann (98) that …“human growth hormone is a heterogeneous protein, consisting of several isoforms” and that. “sources of this heterogeneity reside at the level of the genome, mRNA splicing, post-translation modification, and metabolism.” According to Baumann (98), and especially relevant to this review, we point out that ~50% of hGH isoforms in human blood 15–30 min after a secretory pulse are classified as the 22 kDa monomeric form (half bound to GH binding protein). Oligomeric and aggregated forms are believed to make up a significant portion of the remaining isoforms. Baumann concluded, in 2009 (98), what is true today; viz. that the biological significance of such isoform heterogeneity remains largely unknown. Earlier attempts to purify GH variants (between 1975 and 2000) were directed at understanding their physiological effects; however definitive conclusions relating to their bioactivity remained largely unknown. A review by U. J. Lewis (99) entitled “GH: What is it and what does it do?” makes the point another way. The abstract is provocative and relevant for this review …. “The evidence is now irrefutable that growth hormone (GH), long thought to be a single substance, is actually a mixture of several different forms. These multiple forms must be a consideration in any physiologic study if an accurate evaluation of the actions of GH is to be made” (99).

Fragmentation of the native 22 kDa hormone into two peptides [hGH 1–43] and [hGH 44–191] may affect physiology; the shorter fragment has insulin potentiating activity while the larger has anti-insulin activities, thereby implying that the native molecule acts as a prohormone (99). Similarly, exposure of GH to serine proteases will enhance activity of the hormone at the tibial plate (100). If GH has so many metabolic activities, is their mechanism of action via a common receptor? Lewis addresses this point in a 1996 report: “currently it is believed that all of these actions are mediated through the cloned GH receptor, but this is not proven” (101). To the best of our knowledge that is true to this day.

## Interest in Human Exercise, Bioactive, and Immuno-reactive GH, Re-awakens

Some 20 years after the original report by Ellis et al. (95), pioneering research by Reggie Edgerton, Gary McCall, and Richard Grindeland (at UCLA/NASA Ames) offered evidence for the existence of neural afferent inputs from skeletal muscle that modulated secretion of hGH measured by tibial bioassay. Three trials done between 1995 and 2001 are described in a 2001 review by McCall et al. (102–105). Their designs included: complete bed rest (17 days); astronaut exposure during and after microgravity; and vibration-induced activation of muscle afferents. The exercise component in these trials was either repeated bouts of ankle dorsiflexion or muscle unloading. The interesting findings were that plasma concentrations of bioactive GH changed dramatically, but concentrations of immunoreactive GH were not affected by treatment. These findings clearly challenged the concept that a single molecular form of the hormone is responsible for the growth response (103–105). How activation of a small muscle group, and the neural paths taken, lead to this GH response remains largely unexplored.

How the more standard resistance exercise protocols affected plasma GH, when measured by bioassay and an array of immunoassays, were reported by the Kraemer group between 2001 and 2014. The 2001 study, Hymer et al. (106) was an acute pre-post exercise trial [six sets of 10 at 75% of the 1 repetition maximum (1RM)] involving 35 young (23 year) females tested during the follicular phase of the menstrual cycle. As expected, plasma concentrations of GH, measured by polyclonal, monoclonal radioimmunoassay, and immuno-functional assay [the latter based upon epitope binding of the GH isoform (107), increased after the exercise bout. However, plasma concentrations of GH measured by tibial assay were not different than control samples (Table 2)]. Fractionation of these plasma samples by size exclusion chromatography showed that treatment-induced increases in immunoactive GH was associated with molecular forms in mass ranges expected for dimeric (30–60 kDa) and monomeric (<30 kDa) GH.

**Table 2:** Estimated mean comparisons bioassay, total (BGH) BGH with immunoassay (IGH) concentrations obtained at the same time point from various studies before and after resistance exercise (highest value), and analyses.

**IGH (μg•L**^****−1****^**)**		**BGH (μg•L**^****−1****^**)**		**IGH (μg•L**^****−1****^**) and BGH (μg•L**^****−1****^**) fractions**								
**Rest**	**Post-ex**	**Rest**	**Post-ex**	**≤30 kD rest**	**≤30 kD Post -ex**	**30-60 kD rest**	**30-60 kD Post-ex**	**≥60 kD rest**	**≥60 kD Post-ex**	**Gender**	**Age ± SD (yrs)**	**References**
1.1	1.2	3,800	10,000^*^							Male	43.8 ± 63.8	McCall et al. (103)
Nichols				Nichols(IGH)						Female	23 ± 6.4	Hymer et al. (106)
2.5	9.5^*^			2.5	7.4^*^	2.0	7.5^*^	0.5	1.5			
NIDDK				NIDDK(IGH)								
1.0	2.5^*^			2.5	10.5^*^	1	4.0^*^	0.5	1.0			
				BGH								
		2,200	2,000	1,200	1,000	1,480	1,395	1,400	1,490			
4.1	9.5^*^	1,650	2,400							Female	23.0 ± 1.2	Kraemer et al. (108)
Pre-training										Female	23 ± 3	Kraemer et al. (77)
NIDDK				IGH								*Total-Strength Group*
2.0	3.1^*^			2.0	8.2^*^	1.8	4.8^*^	0.2	0.8			
Nichols												
2.5	11.3^*^			3.1	8.0^*^	3.0	9.1^*^	1.0	2.0^*^			
				BGH								
		2,450	3,150	1,500	650^*^	990	750	1,400	4,150			
Post-training												
NIDDK				IGH								
3.2	7.0^*^			4.8	12.0^*^^#^	2.5	8.8^*^^#^	0.8	1.5			
Nichols												
4.8	14.2^*^			2.0	6.1^*^^#^	2.5	10.0^*^	0.8	1.0			
				BGH								
		3,850^#^	3,450	1,250^#^	1,500^#^	1,250	1,250^#^	1,150	2,450^*^^#^			
Pre-training										Female	26.3 ± 4.0	Kraemer et al. (77)
NIDDK				IGH								*Total-Hypertrophy Group*
1.8	2.5^*^			2.6	8.0^*^	1.2	3.8^*^	0.1	0.8			
Nichols												
2.7	8.0^*^			2.7	8.2^*^	1.6	7.2^*^	0.3	2.0^*^			
				BGH								
		2,950	1,900^*^	1,550	1,010	1,650	1,100	1,950	1,550			
Post-training												
NIDDK				IGH								
1.8	4.5^*^			2.4	7.0^*^^#^	2.0	4.8^*^	0.2	0.8			
Nichols												
1.9	13.1^*^^#^			1.1	5.0^*^^#^	1.3	8.6^*^^#^	0.1	1.2^*^			
				BGH								
		2,900	2,500^#^	1,090	1,190	1,950^#^	750^*^	1,600	2,010^#^			
				%	%	%				Female	61.6 ± 1.3	Gordon et al. (110)
Old				IGH	IGH	IGH						*Resistance Ex*
2.5	4.8^*^		980	30	55	15						
				BGH	BGH	BGH						
				15	45	40						
Young				IGH	IGH	IGH						
3.5	17.5^*^		1,725	40	40	20						
				BGH	BGH	BGH						
				30	40	30						
1.0	10.0^*^	6,400	11,500							Male	20.1 ± 2.1	Thomas et al. (109)
0.4	7.0^*^	3,800	6,200							Male	21.0 ± 2.1	Thomas et al. (109)
4.5	16.5^*^		1,740							Female	23.7 ± 1.0	Gordon et al. (110)
0.6		2360.9								Male	80.5 ± 1.6	Kraemer et al. (1)
2.0		4966.1								Female	80.7 ± 1.4	Kraemer et al. (1)

* *Significant increase from corresponding resting value*.

# *Significant difference from pre-training*.

From the results of this initial 2001 study (106), which involved untrained women, it was clear that the pituitary failed to respond to the exercise stress by secreting additional biologically active GH. To address the question of possible importance of exercise training, Kraemer et al. (111) undertook an extensive 6 month training program using different combinations of resistance training (i.e., either total body or upper body) using a progressive linear periodized training program supplemented by standard endurance training. As expected, each of the training groups experienced significant gains in the strength of the involved musculature over the training period, thus lending internal validity to the training study. Plasma samples were obtained both pre- to post- resistance exercise and pre- and post-training. With training, and as expected, iGH concentrations increased even further and highest assay signals were recorded using monoclonal antibody. bGH concentrations in both unfractionated and fractionated plasma samples were variable with four different training groups (two total body training groups presented in Table 2), In this same trial, GH assays of form(s) contained in three molecular weight classes, prepared by size exclusion chromatography, yielded equally interesting results. Thus, smaller (30 kDa) molecular mass variants generated the largest immunoreactive responses; however, larger (>60 kDa) molecular mass variants contained form(s) that were equally as potent as the small (30 kDa) and medium (30–60 kDa) class fractions in terms of generating a bone growth response. We believe this interesting result reflects the importance of either di-sulfide linked GH aggregates, and/or GH bound to GH-binding protein, for generation of somatogenic activity.

But most important exercise-induced changes in GH bioactivity were experienced after 6 months of training (6 × 10 squat at 80% of 1 RM with 2 min rest between sets).

- The total body strength training group demonstrated in the unfractionated total a significant elevation in resting bGH, and with training in the >60 kD fraction showing uniquely an increase with acute exercise and this acute response was significantly higher post-training. Additionally, other fractions also demonstrated higher post training values. Thus, the bGH appeared for the first time to be responsive to exercise stress and also demonstrated adaptations to training in these young women. While not shown in Table 2 the upper body only strength training group also showed similar changes with significant increases in the unfractionated resting values as well as a significant exercise-induced response following training, again showing the influence of training on bGH.
- In the total hypertrophy group, it was observed that pre-training acute exercise resulted in a significant decrease in the UF samples and this was observed again post-training yet the post-exercise values were significantly higher. Again, while not shown in the table, the upper body group showed no acute exercise changes in the UF samples pre-training but with training, resting values were significantly higher and a significant exercise-induced elevation was observed.

Taken together, these results indicated for the first time that acute and chronic exercise training using conventional large muscle group resistance training protocols will increase (acutely and chronically) plasma concentrations of GH bioactivity in young women. McCall had shown previously that exercise of small muscle groups would also increase plasma concentrations of bGH (103, 104).

Data from other studies also reveal the dichotomy between bioactive and immunoreactive GH. Comparison of bGH plasma levels from 24 vs. 62 year old female volunteers, after acute aerobic cycle exercise, were not different. However, after an additional acute resistance exercise bout, plasma concentrations of bGH from the younger group were significantly higher than those in the older group. These higher concentrations were associated with molecular forms of apparent mass 30–55 kDa (i.e., dimer range) (110).

Comparison of bGH plasma concentrations from lean [BMI = 23] vs. obese [BMI = 36] men revealed that although resistance exercise had no significant effect, their concentration in the leaner group was significantly higher. Similar to other studies, concentrations of GH measured by immunoassay were not different between the two groups (109).

A trial done with free-living 81 year old individuals, failed to uncover differences in plasma GH concentrations (measured by either bioassay or immunoassay) that could be correlated with either fitness or physical performance. Curiously, one half of the group (*n* = 21) had plasma concentrations of bioactive GH that were essentially zero, while the other half (*n* = 20) had concentrations that were readily detectable and in the range of studies listed previously (112).

## How Experiments With Rats Offer Clues Relevant to Human Exercise and Bioactive GH

### Somatotroph Heterogeneity

Cell separation studies indicate that two populations of GH cells (somatotrophs) are present, in roughly equal numbers (~40%), in the rat pituitary gland. One population (light somatotrophs, also designated the type I cell) has densities <1.071 g/cm^3^, while the other (heavy somatotrophs, also designated the type II cell) has densities in ranges >1.071–1.085 g/cm^3^. The higher density of the type II cell is attributable to large numbers of 300 nm diameter, GH containing, cytoplasmic secretory granules. Results from a recent experiment (113), designed to determine if the GH released from light vs. heavy somatotrophs is differentially active by bioassay, offer definitive evidence to support the hypothesis that differential responses between bioassay vs. immunoassay results after human exercise (described previously), has a structural (cellular) basis residing within the pituitary gland itself. Results of this experiment showed that: (1) culture media from type II cells contained 5x as much bGH (tibial assay) as that from type I cells; (2) net production of bGH from type II cells was 6x more than that from type I cells (*p* < 0.001), but production of iGH was not different between type I vs. II cells; (3) implantation of type II cells into rat brain ventricles of hypophysectomized recipients significantly increased body weights, tibial widths and gastrocnemius muscle; however, implantation of type I cells had little to no significant effect on these same markers; and (4) type II cells prepared from animals that had been previously fasted or insulin injected showed markedly reduced bGH secretion. Recent studies using RNAseq assays also demonstrate somatotroph heterogeneity in mice, e.g., a subpopulation enriched in sterol/cholesterol synthesis genes (114). Additionally, another study using RNAseq assays also showed a subpopulation of somatotrophs demonstrating sex dependent differences in anterior pituitary cells in female rats (115), Thus, others have also found somatotroph heterogeneity using other molecular techniques harking back historically to some of the first observations of this phenomenon (116, 117).

### The GH Secretory Granule

These membrane bound cytoplasmic organelles contain ~75% of the total bioactive hormone measured in the pituitary homogenate (118). The hormone in the granule is bound cooperatively with two Zn(II) ions per GH dimer (119). Each granule is estimated to contain 5,000–10,000 molecules and its dense core consists of large, crystal-like aggregates which are thought to solubilize on exocytosis (120–122). Some GH granules contain cytochrome C, cytochrome oxidase and ATP; molecules that may mediate GH release (121).

On electrophoresis in non-reducing SDS gels, rat pituitary extracts contain a wide range of di-sulfide linked GH variants (14–88 kDa MW) (123). Electro-elution of protein from different regions of such gels, followed by their chemical reduction, apparently uncovers epitopes hidden in the aggregate, thereby increasing iGH activity up to 6X. Oligomeric forms >44 kDa are found exclusively in extracts prepared from dense, highly granulated, purified type II- bGH producing- somatotrophs (pentamers). Extracts from the less dense, less granulated, type I somatotrophs contain a single dominant 22 kDa peak and a minor 44 kDa species (dimer). Chemical reduction of culture media from type II, but not type I, somatotrophs increases immunoreactivity (5X vs. 1.3X, respectively). This important result confirmed maintenance of granule heterogeneity within the somatotroph in cell culture. Since GH released from the type II somatotroph, relative to type I cells, is most active in both *in vitro* (cell culture) and *in vivo* (hollow fiber implant) bGH tests, the results of Farrington and Hymer (123) and Grindeland et al. (113), support the contention that bGH activity is associated with disulfide linked aggregates (oligomers) residing in granules of the type II somatotroph, as well as bGH activity in culture media secreted from the type II somatotroph.

### Growth Hormone Is Stored as an Amyloid

A major advance in understanding packaging mechanisms of GH molecules within a secretory granule came from the reports of Maji and co-workers showing that the hormone is stored as an amyloid (124, 125). Amyloids are defined by their highly organized cross B-sheet regions in protein aggregates and should be considered as yet another level of protein structure. The cross-B sheet represents a single structural epitope in which individual strands of each sheet run in perpendicular to the fibril axis while B-sheets are parallel to the fibril axis. These highly organized, elongated amyloid fibers are composed of thousands of copies of stacked B sheets composed of peptide/protein. These stacked fibers can trigger further refolding of the natively folded protein. In many proteins the amyloid state is thermodynamically stable at high concentration, but not energetically favorable at lower protein concentration (126). These fibrillary structures are often hallmarks of severe disorders; e.g., Alzheimer disease and diabetes mellitus.

Amino acid residues 72–82 of the 191 amino acid, 22 kDa rHGH monomer have a high aggregation propensity and 4 fibrillation segments, each of ~6–10 residues. These are B aggregation “hot spots.” Only Zn(II) ion, as the specific helper, allows fibrillation; yet even in this configuration, most of the molecule is able to maintain its globular fold (125)! The amyloid configuration not only may ensure efficient release of 22 kDa GH from the amyloid depot, but also protect the GH from enzymatic degradation, high temperature, and large pH ranges. It is now well-accepted that many proteins can assume the amyloid configuration.

Mechanisms underlying amyloid fibril formation, and their relationships/ interactions (sometimes reversible) leading to the formation of either disordered, amorphous aggregates or oligomers (via on/off pathways), will lead to varied configurations of amyloid. These conformations are complex, dynamic and thought critical for understanding protein configuration in health and disease (126). Many proteins form amyloid-like fibrils *in vitro*. Obviously not all proteins are “bad.” It must be recognized that common structural principles of amyloids convey their double nature as “good” or “bad” (127).

**Figure 2:**
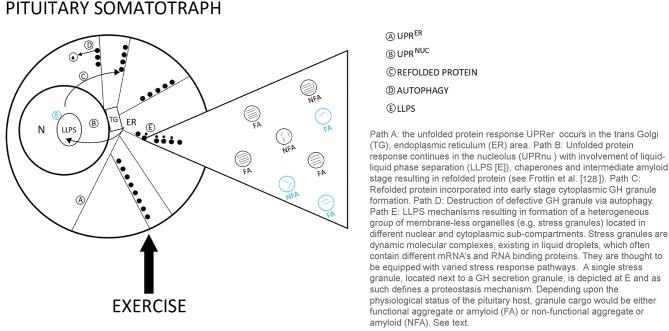
Theoretical model of proteostasis mechanisms likely to be active in the pituitary somatotroph during various types of human exercise stress.

In the resting state, GH synthesis and processing of functional molecular aggregates (FA) [“good aggregated GH”] follow the regulated path to the cell surface and become primed for stimulated secretion into the blood. As the demand for GH increases with exercise stress, this process may result in errors in the biosynthetic pathway. Mechanisms to repair mis-folded, non-functional GH aggregates (NFA), are shown in Figure 2. As summarized by Frottin et al. (128) it has also become apparent that the nucleolus plays and important function in maintaining the homeostatic (proteostasis) quality control of aggregated proteins in the cell to prevent the formation of toxic aggregates or what might also be called non-functional aggregated proteins arising from aberrant cellular processing. Some NFA forms could be released into the circulation, however the concentration of circulating FA and NFA forms remains largely unknown. These repair mechanisms may be enhanced with exercise training. This model suggests an intriguing line for future research in the quest to understand roles of aggregated GH in stress biology (129–131). The potential for lower values of BGH in the blood might be observed if all of the processing systems for mis-folded non-functional GH aggregates are fully engaged, potentially a training adaptation.

### Acute and Chronic Exercise Complexity Remains

While the GH responses to exercise has been characterized for decades understanding the many selective roles in metabolism and other physiological mechanisms related to acute homeostasis and repair and remodeling of tissues remain needed (94, 132–136). It becomes apparent that understanding the role(s) of GH in responding to exercise stress and adapting to exercise training is still in its embryonic stage. This becomes evident when one realizes that GH is not a single entity. The multitude of roles attributed to GH require that a more complex set of mediating mechanisms may be needed to accomplish them. As noted in this section the diversity of GH isoforms from their presence in the anterior pituitary to other biocompartments (e.g., brain, circulation, liver) also suggest that target cells may be responding to different GHs. The mere differences in receptor binding between bio and immune assays and their differential signaling raise questions as to their acute and chronic roles in exercise stress and adaptations. Additionally, growth hormone binding proteins from the liver and their potential to create dimers when binding to 22 kD forms in the blood also raise questions as to how they function in signaling (137–139), yet while increases with acute resistance exercise are observed differences between trained and untrained men have not been observed (140).

Types of exercise may well have an influence as well (63). It may be due to total amount of work or the inability to activate the same motor unit array that contributes to such modality differences. One unifying thought is the influence of pH and H+ ions on IGH (141). This is reflected in its close associations of blood lactate, that when lactate is elevated beyond the anaerobic threshold or is dramatically elevated with a resistance training workout, IGH is highly responsive (134, 142–145). This was demonstrated with resistance exercise in men and women in two studies by Kraemer's research groups (144, 145) where the short rest workouts using 1 min between sets and exercises demonstrated the highest blood lactate responses and IGH responses. Whether this is due to a reduction in the type 2 somatotrophs production less aggregate or a stimulation of predominantly type 1 somatotrophs is unknown. Other factors such as body fat of subjects to fasted or intakes of protein/carbohydrate before and/or after the workout also appear to influence IGH. Since the BGH studies have always been done in the fasted state, nothing is known as to its response patterns. Additionally, with the stability of the BGH in the blood how pulsatility of IGH interfaces with the entire signaling milieu remains to be elucidated.

Finally, how the various splice variants and aggregates of GH are integrated within the larger web of hormonal and molecular signaling remains to be seen as various studies continue to unravel the complex nature of homeostatic regulation with acute exercise and chronic exercise adaptations.

## Current Perspectives on IGF-I and Physiological Adaptations and Complexity Related to this Superfamily to Training

Insulin-like growth factors (IGFs) are small polypeptide hormones (70 and 67 amino acids for IGF-I and IGF-II, respectively), structurally related to insulin, and synthesized from a larger precursor peptide that is post-translationally processed into its active form. Of the two, IGF-I has been most extensively studied and is secreted as it is produced by the liver in response to GH stimulation. Only 2% of IGF-I circulates in its free form; most circulates as a binary (20–25%) or ternary complex (~75%) (146–149). In its binary form, IGF-I circulates with one of seven binding proteins whereas in its ternary form, IGF-I circulates with IGFBP-3 and its acid labile subunit (ALS).

IGF-I (7 kDa) is responsible for metabolic, mitogenic and anabolic cellular responses (150). It is produced locally (i.e., autocrine and paracrine mechanisms) in tissues and cells. IGF-I acts as both a cell cycle initiation and progression factor. Its effects include satellite cell activation, proliferation, survival, and differentiation, increasing myotube size and number of nuclei per myotube, stimulating amino acid uptake and protein synthesis and muscle hypertrophy, neuronal myelinization, axonal sprouting and repairing damage, reducing chronic inflammatory response, increasing free fatty acid utilization, and enhancing insulin sensitivity upon receptor binding and subsequent intracellular signaling and glucose metabolism (1, 151). Expression and secretion of IGF-I increases by myofibers with mechanical loading (152). Secretion by myofibers stimulates autocrine and paracrine myofiber anabolic processes where adjacent satellite cells enter the cell cycle, proliferate, differentiate, fuse with myofibers, and provide myonuclei to maintain or reestablish the myonucleus to myofiber size ratios of the enlarged myofibers (152). Because of these critical anabolic functions, IGF genes have been considered a potential target for gene therapies, gene doping in athletes (153) and staving off advancing muscle weakness (154).

While liver-derived IGF-I is under direct regulation of GH, local mechanical-stretch mechanisms can activate IGF-I synthesis in tissues. The potency of circulating IGF-I remains unclear and needs to be viewed in context with its binding proteins that provide fine tuning of the IGF actions and regulate bioavailability (150). Several studies have shown systemic elevations in IGF-I produced no elevations in protein synthesis or hypertrophy during resistance exercise training whereas up-regulation in the muscle isoform was linked to significant muscle hypertrophy (151).

## Acute Responses and Chronic Adaptations of IGFs to Resistance Training

There remains much to discover about the roles of systemic vs. locally produced IGF-I in mediating the outcomes of resistance exercise (155). Yet, it appears that local IGF-I is consistently upregulated with both acute and chronic exercises; whereas in certain situations, circulating IGF-I may actually decrease, increase, or not change (21, 155). Studies showing no change in circulating IGF-I can vary due to the temporal frame of measurement following stimulation with GH (21). While the acute responses of IGF-I have been evaluated in the serum/plasma of many different studies of resistance exercise, its contribution to hypertrophy has been difficult to determine due to the milieu of anabolic signaling to skeletal muscle. Kraemer et al. were the first to demonstrate this highly variation to resistance exercise stress of IGF-I (119). However, there is little doubt, IGF-I is a primary player in anabolic signaling targeted to many tissues, including skeletal muscle. It could be that IGF-I acts as a signal that either amplifies or regulates skeletal muscle tissue repair and remodeling (1). Looking at the IGFBPs has provide a more fruitful area of study as they have shown a more reliable pattern of responses to acute resistance exercise protocols. Of importance is the response of IGFBPs which have generated more consistent responses with resistance exercise acutely elevating IGFBP-3 (21). Looking on longer term changes in IGF-I, Nindl et al. (148) monitored overnight IGF-I following heavy resistance exercise and showed IGF-I concentrations remained unaffected. However, IGFBP-2 increased and ALS decreased indicating that binding protein partitioning, rather than changes in systemic IGF-I, appeared to be an important finding. Exercise duration and total work also may impact IGFBP-1 changes but it was not see that the modality had as much impact on the response patterns. With the novel technique of microdialysis to measure IGF-I in the interstitial fluid, Nindl et al. (149) showed total and free IGF-I and IGFBP-3 were elevated. However, IGF-I in interstitial fluid was unaltered following high-power resistance type exercise. It was also observed that the IGF-I receptor phosphorylation was not increased but IGF mRNA content and Akt phosphorylation were increased (149) This supported the speculation that skeletal muscle adaptation is not be directly dependent on systemic IGF-I, but rather be involved with the interactions and signaling across different biocompartments.

Long term resistance exercise training studies examining resting circulating IGF-I concentrations have been demonstrated to be highly variable with reductions, no change, and elevations with no change or reductions in IGFBP-1 and IGFBP-3 (21). It has been demonstrated that in participants who are classified as extreme responders to a long term (16 wk) training program showed no significant changes in IGF-I, IGFBP-1, or IGFBP-3 but a trend showed that IGFBP-3 was lower in the non-responders (156). Resistance-trained men have been shown to have higher resting IGF-I values than untrained men (140) Nevertheless, single measurements of IGF-I need to be carefully interpreted as the roles and contributions remain speculative due to the multiple targets and mechanisms they are involved with in the signaling processes. Of more consequence may be the training responses of locally-produced IGF-I isoforms. Resistance exercise training of sufficient intensity and volume increases IGF-I and MGF mRNA for up to 48 h post RE (21, 157). Furthermore, IGF-I and MGF mRNA have increased 2 h post exercise (but not 6 h) after a single bout of moderate (65% of 1RM; 18–20 repetitions) and moderately-high (85% of 1RM; 8–10 repetitions) intensity resistance exercise training (158). Further studies have shown MGF acts independently and is expressed earlier than other IGF-I isoforms in response to resistance exercise training, and therefore may have greater anabolic potency (159). The recruitment of motor units and their associate muscle fibers creating mechanical damage appears to be an essential stimuli for local production of IGF-I.

## IGF-I Receptor and Intracellular Signaling

Downstream actions of IGF-I are mediated through binding to the IGF-I receptor (IGF-IR), a ligand-activated receptor tyrosine kinase on the cell surface of target tissues. The IGF-IR gene is mapped to chromosome 15q25-26. Activation of receptor tyrosine kinase activity results from ligand binding to the α subunit of the receptor leading to a conformational change in the β subunit (160). This leads to the activation of downstream signaling pathways of IGFs including PI 3-kinase pathway and Ras-mitogen-activated protein kinase (MAP kinase) pathway, for cell proliferation, cell differentiation and cell survival (160). Two types of IGF receptors have been identified. The type I receptor binds IGF-I with greater affinity than IGF-II and also interacts weakly with insulin. The type II receptor binds with greater affinity to IGF-II than IGF-I and does not bind to insulin (161). Resistance exercise influences IGF-IR phosphorylation where high-volume results in greater phosphorylation compared to high-intensity protocols 1 h post exercise (162). Resistance exercise protocols of moderate to high intensity also have been shown to increase IGF-IR mRNA 2 h following acute exercise (158). Mechanical stress also stimulates IGF-R signaling cascades via focal adhesion kinase (FAK), an attachment complex protein necessary for mechanical IGF-I-mediated hypertrophy in skeletal muscle cells (163). To the contrary, anabolic resistance and sarcopenia may be attributed to dysregulation in the IGF stimulated, Akt /Protein Kinase B and mechanistic target of rapamycin (mTOR) signaling pathways in response to resistance exercise and protein intake (164).

## Integrated Communication for Anabolic/Catabolic Signaling: Glucocorticoids

### Cortisol Regulation

**Figure 3:**
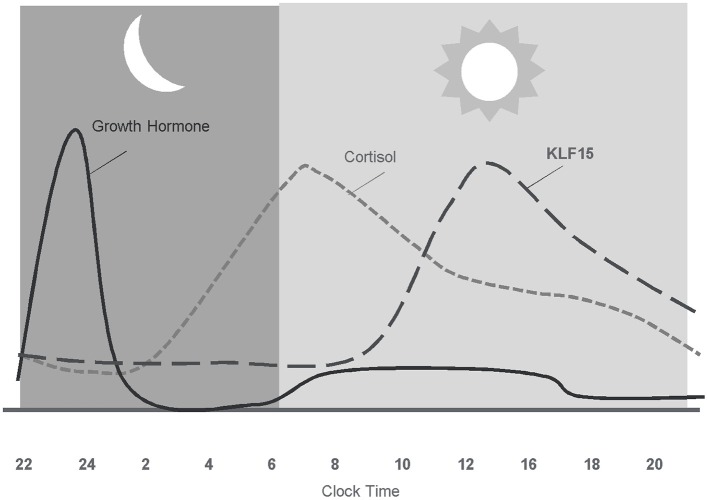
Diurnal pattern of anabolic/catabolic regulators may facilitate anabolic benefit of intermittent exposure.

In addition to the anabolic hormones, glucocorticoids, mainly cortisol have a profound influence on human skeletal muscle (165). During stable physiological conditions, circulating cortisol exhibits a circadian rhythm peaking in the morning, slowly decreasing throughout the day, and reaching lowest levels around midnight (166) (Figure 3). Cortisol levels are regulated both at the systemic and tissue level to maintain glucocorticoid homeostasis. Endogenous levels of cortisol are systemically controlled by the hypothalamic-pituitary-adrenal (HPA) axis and locally by the action of 11β-hydroxysteroid dehydrogenase (11β-HSD) enzymes. In the periphery, the cellular response to glucocorticoids differs by cell type (167–169), cell cycle stage (167), and exposure to stress (170).

In skeletal muscle, cortisol plays a fundamental role in regulating energy homeostasis and metabolism (171). During exercise, cortisol increases the availability of metabolic substrates, protects from immune cell activity, and maintains vascular integrity (172). The acute cortisol response to exercise is highest when the overall stress (volume and/or intensity of total work) of the training period is high (145, 173). Cortisol is also involved in adaptations to exercise by preparing the body for the next bout of exercise (71, 174), as increases in cortisol are prolonged before returning to basal levels following a bout of exercise. Adaptation of the HPA axis following exercise training is largely manifested by altered sensitivity to cortisol (172). Following acute exercise, there is an increased tissue sensitivity to glucocorticoids that serves to counteract muscle inflammation, cytokine synthesis, and muscle damage (172). Subsequent decreased sensitivity of monocytes to glucocorticoids 24 h following exercise may act to protect the body from prolonged, exercise-induced cortisol secretion (172). Inactivation of cortisol into cortisone acts as another mechanism to protect tissues and cells from the deleterious effects of exercise-related cortisol secretion (175). Inactivation of cortisol to cortisone appears to be an adaptation to exercise, given that athletes display a higher inactivation of cortisol into cortisone (175). However, overtraining appears to impair the inactivation of active cortisol to cortisone in athletes (175), and may impair anabolic processes as high levels of cortisol decrease skeletal IGF-I synthesis by reducing IGF-I transcript levels (176).

## Tissue Specific Regulation BY 11β-HSD (11β-hydroxysteroid Dehydrogenase)

11β-HSD (11β-hydroxysteroid dehydrogenase) acts as a tissue specific regulator of glucocorticoid action by catalyzing the interconversion of active cortisol and corticosterone with inactive cortisone and 11-dehydrocorticosterone (177). This interconversion regulates glucocorticoid access to intracellular glucocorticoid receptors (178) and glucocorticoid action (179). The cellular hormonal environment can influence 11β-HSD activity, where exposure to insulin, insulin-like growth factor I, and glucocorticoids can alter enzyme activity (179). Raised expression of 11β-HSD1 (Type 1) in skeletal muscle is believed to play role in mechanisms that contribute to the development of metabolic syndrome (180) insulin resistance (181), and hypertension (182).

## Glucocorticoid Receptors

Glucocorticoids convey their signal mainly through intracellular glucocorticoid receptors, which in the absence of a ligand are generally localized to the cytosol (183). In the cytoplasm, the glucocorticoid receptor is found in a complex with chaperone proteins that maintain a conformation with high affinity binding potential (89). Once a glucocorticoid binds to the receptor, it moves to the nucleus where it interacts with specific DNA sequences known as glucocorticoid response elements (183, 184). Glucocorticoid response elements regulate the transcription of primary target genes by either directly binding to DNA (185), tethering onto other DNA-binding transcription factors (185), or through direct protein-protein interactions with other transcription factors and/or coregulators (186). Glucocorticoid receptor-binding to DNA is highly context specific and relies on the interplay of the receptor with other proteins (187, 188).

Selective targeting of glucocorticoid receptors is mediated by the combined action of cell-specific priming proteins, chromatin remodelers (189), and local sequence features (190). As much as 95% of glucocorticoid receptor binding sites are within preexisting sites of accessible chromatin (190), with some detected in remodeled chromatin (189, 190). Binding is dictated by proteins that maintain chromatin in an open state (188). Activator protein 1 (AP1) is one such protein that is involved in glucocorticoid receptor chromatin interactions and subsequent transcription and recruitment to co-occupied regulatory element (188). Most (62%) GR-binding sites are occupied by the transcription factor C/EBPβ (enhancer-binding protein beta) (189), which regulate multiple genes in the ubiquitin-proteasome pathway (191).

During myogenesis, glucocorticoid receptors are localized in different parts of cells: in the cytoplasm of myoblasts, in the nucleus of myotubes, and in the extracellular matrix, satellite cells, and near mitochondria in mature skeletal muscle fibers in mice (192). Yet, location may differ by fiber type, as most muscle fiber types express glucocorticoid receptors in the cytosol, but only slow fibers express glucocorticoid receptors on the membrane (193). Membrane glucocorticoid receptors are localized in the extracellular matrix and signal rapidly (within 5 min) through the MAPK pathway in mammalian skeletal muscle fibers (192).

### Glucocorticoid Receptor Isoforms

**Figure 4:**
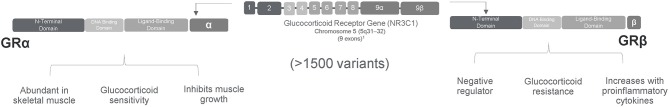
Alternative splicing of a single gene results in two major isoforms of glucocorticoid receptor with more than 1,500 variants.

The human glucocorticoid receptor is encoded by the NR3C1 gene, located on chromosome 5 (5q31–32) (194), and consists of nine exons (195). There are two major isoforms of glucocorticoid receptor due to alternative splicing of a single gene: GRα and GRβ (196). These isoforms differ at their carboxyl termini (195) (Figure 4). GRβ has a truncated glucocorticoid ligand-binding domain, which prevents glucocorticoid binding and causes GRβ to act as a dominant negative inhibitor of GRα (195, 196). In healthy humans, the default splicing pathway is the one leading to GRα (197), with minimal activation of the alternative splicing event leading to GRβ (197). While there are two main isoforms of the glucocorticoid receptor, more than 1,500 variants have been identified and cataloged (198). Such variants include both naturally occurring and stress-induced GR isoforms, where further studies are needed to decipher their roles in stress responses (198). In healthy human cells and tissues, GRα mRNA concentrations are highest in the brain, followed by skeletal muscle, macrophages, lungs, kidneys, liver, heart, eosinophils, peripheral blood mononuclear cells, nasal mucosa, neutrophils, and colon (197). GRβ mRNA expression which is lower than GRα mRNA expression, with the highest concentrations found in eosinophils, followed by peripheral blood mononuclear cells, liver, skeletal muscle, kidney, macrophages, lung, neutrophils, brain, nasal mucosa, and heart (197).

The relative expression of the two alternatively spliced glucocorticoid isoforms and the ratio of GR-α to GR-β expression modulates cellular sensitivity to glucocorticoids (199). Expression of GRβ selectively increases in cells exposed to inflammatory signals; this increased expression leads to glucocorticoid resistance (196, 200) and may reduce the therapeutic potential of glucocorticoids (201). In myoblasts, glucocorticoid exposure results in a dose-dependent decline in GRα expression and a dose-dependent increase in GRβ expression (179). In myotubes, overexpression of GRβ is associated with a blunted catabolic response to glucocorticoids via lower “atrogene” signals (201). Mechanistically, the selective increase in GRβ appears to involve the splicing factor SRp30c (serine/arginine-rich protein p30c) (202, 203). On the other hand, agents that increase GRα expression sensitize cells to glucocorticoids (204). Exercise affects receptor expression (205) and relative expression of receptor isoforms; athletes show less GRα mRNA expression in peripheral blood mononuclear cells than do untrained controls, indicating reduced sensitivity (206). Yet, GR-β does not appear involved in exercise adaptations in peripheral blood mononuclear cells of athletes (206).

### GRα Isoform Signal

**Figure 5:**
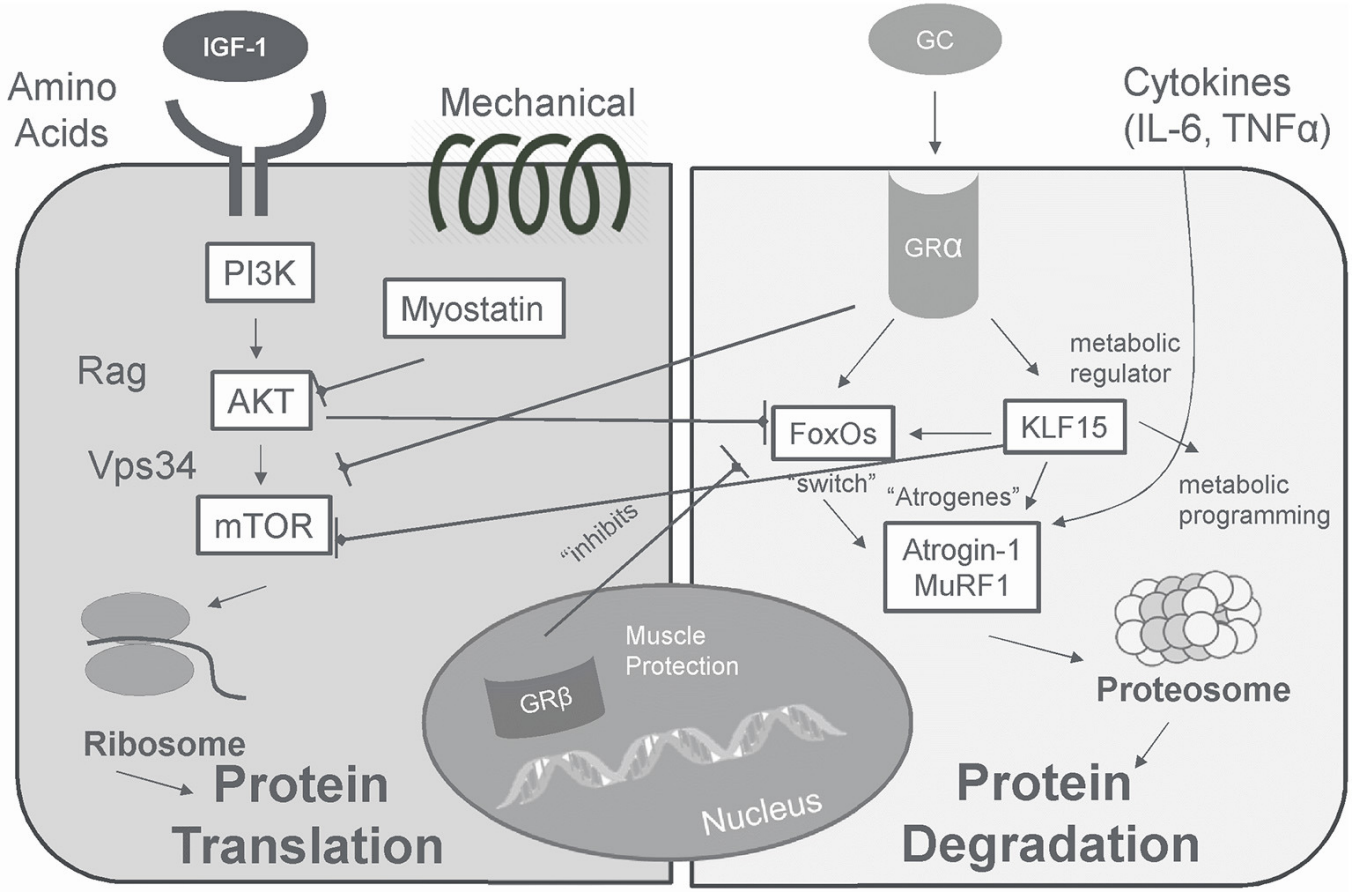
In skeletal muscle glucocorticoids produce a catabolic effect that is opposite that of insulin/IGF-I via GRα.

In skeletal muscle, glucocorticoid hormone action is determined principally by binding to the GRα isoform (179) which can increase or decrease glucocorticoid receptor gene products that contribute to physiologic responses (207) (Figure 5). The binding of glucocorticoids to the ligand-binding domain of GRα causes translocation to the nucleus and binding to glucocorticoid response elements (GREs) in the promoter region of genes (201). Specifically, GRα binds to GREs in the promoter of forkhead box O (FOXO) transcription factors and enhances expression (208). This results in a FOXO-dependent increase in muscle atrophy F-box/Atrogen-1 (MAFbx) and muscle ring finger 1 (MuRF1), E3 ubiquitin ligases necessary for glucocorticoid -induced muscle myopathy; suppression of MAFbx and MuRF1 inhibits glucocorticoid -induced protein degradation (208). In addition, glucocorticoids may also exert actions through tethering (GR binding to other transcription regulators) and squelching (GR binding to and taking away transcription regulator from DNA), which often lead to transcription repression (185).

### Proteolysis Signal

The catabolic actions of cortisol resulting in muscle proteolysis occur largely via the ubiquitin–proteasome and lysosomal systems (186, 209–211). Via these proteolytic systems, expression of genes involved in atrophy (“atrogenes”) are increased, which target proteins for degradation by the proteasome machinery (210). Atrogenes include transcription factor FOXO, a major switch for the stimulation of several atrogenes, and two ubiquitin ligases atrogin-1 and MuRF-1, involved in the targeting of protein to be degraded by the proteasome machinery, and LC3 (186, 201, 209, 210). Glucocorticoids also may blunt skeletal muscle protein synthesis by inhibiting IGF-I signaling, a muscle anabolic growth factor, and increasing myostatin signaling, a muscle catabolic growth factor, contributing to muscle atrophy (207, 209, 210).

### GR Receptor Expression in Skeletal Muscle

In skeletal muscle, glucocorticoid receptor expression is more abundant in fast than slow twitch fibers (211, 212). Consequently, slow twitch muscle fibers appear to be resistant to the catabolic action of glucocorticoids (213) whereas, fast twitch muscle fibers are more sensitive to the catabolic action of glucocorticoids (214). Glucocorticoid-induced muscle catabolism results from degradation of contractile proteins which begins in the myosin filaments and then spreads to the thin filaments and the z-line (213). In fast fibers, glucocorticoid exposure in the absence of exercise increases the activity of non-lysosomal proteases (214). Yet, in response to exercise, both fast and slow fibers experience increases in myofibrillar protease activity followed by anti-catabolic actions (214). While GR expression does not appear to change following resistance exercise (76), receptor activation occurs at a rate that is independent of both fiber type and delivery of steroid to working muscles during exercise (215).

### GRβ Isoform Signal (Negative Regulator)

GRβ functions as a negative regulator of glucocorticoid actions in local tissues (168), where overexpression of GRβ is associated with glucocorticoid resistance. Like other nuclear receptors, the GRβ functions as a naturally occurring dominant negative isoform that blocks the activity of GRα when the two are co-expressed in the same cell (195, 216). The negative action is largely caused by the formation of inactive, or weakly active, heterodimers between GRα and GRβ (216, 217). Unlike the GRα, GRβ has a truncated ligand-binding domain that prevents glucocorticoid binding and causes glucocorticoid resistance (195, 201). The dominant negative activity of GRβ resides within its unique carboxyl-terminal 15 amino acids (217). In addition, unlike GRα, GRβ is located primarily in the nucleus of cells independent of hormone administration (195). In the absence of GRα, GRβ is transcriptionally inactive on a glucocorticoid-responsive enhancer (195). When both GRα and GRβ isoforms are expressed in the same cell, GRβ inhibits the hormone-induced GRα -mediated stimulation of gene expression (195). Compared to GRα, GRβ does not undergo ligand-induced down regulation and has an increased half-life (195). Elevated levels of GRβ in immune cells correlate with reduced sensitivity to glucocorticoids (168). Expression of GRβ in cells is increased by proinflammatory cytokines [interleukins IL-1, -2, -4, -7, -8 and -18; tumor necrosis factor -alpha (TNFα); and interferons α and γ] (168, 200).

GRβ is responsible for the development of tissue-specific resistance to glucocorticoids in various disorders associated with dysregulation of immune function (168). Increased GRβ expression has been linked to glucocorticoid resistance in asthma, leukemia, cancer, and inflammation (201). GRβ expression in human neutrophils may also provide a mechanism by which cells escape glucocorticoid-induced cell death (218). Cell survival is further enhanced by upregulation of GRβ by proinflammatory cytokines such as IL-8 in the presence of glucocorticoids during inflammation (218). Anti-GRβ molecules have become a target of cancer therapies as GRβ has been shown to be highly expressed in cells from solid and liquid tumor, and blocking them may repress cell migration (219). On the other hand, GRβ may serve as a pharmacological target for skeletal muscle growth and protection from glucocorticoid-induced catabolic signaling (201). Increased expression of GRβ promotes glucocorticoid resistance in skeletal muscle, thus stabilizing muscle mass during exposure to high doses of glucocorticoids (201).

Muscle protection via GRβ is associated with increased levels of muscle regulatory factors, enhanced proliferation in myoblasts, and increased myotube fusion (201). Myotubes overexpressing GRβ have lower forkhead box O3 (FOXO3a) mRNA levels and a blunted muscle atrophy F-box/atrogen-1 (MAFbx) and muscle ring finger 1 (MuRF1) response to glucocorticoids (201). GRβ also enhances insulin-stimulated growth through suppressed phosphatase and tensin homolog (PTEN) gene expression and increased phosphorylation of Akt (220). Moreover, overexpression of GRβ may preserve skeletal muscle mass in the presence of glucocorticoids by increased MyoD (1.8-fold) and myogenin (2.5-fold) gene expression, two muscle regulatory factors necessary for skeletal muscle development and regeneration (201). In addition, overexpression of GRβ enhances myotube formation and reduces glucocorticoid responsiveness in mouse muscle cells (201). Another protective mechanism by which GRβ contributes to preserved muscle mass may be through repression of the tumor necrosis (TNF) α and interleukin (IL)-6 genes (221), and inhibited GRα -mediated repression of an NF-kappaB-responsive promoter (217). Yet, glucocorticoid exposure alone does not appear to impact GRβ protein levels in mouse muscle cells (201) and human cells (222).

To the contrary, insulin exposure increases GRβ protein expression (201). Thus, insulin resistance in response to glucocorticoid therapy may contribute to muscle atrophy via reduced protein synthesis and increased protein degradation by genomic and non-genomic interference with several kinases in the insulin-signaling pathway (201). Although further work is needed to determine the impact of physical exercise training on GRβ, studies in human myoblast and myotube cultures (without neural innervation, mechanical loading, and *in vivo* conditions) revealed that treatment with glucocorticoids alone may not be sufficient to elicit changes in GRα or GRβ mRNA or protein expression (222).

### Glucocorticoid Sensitivity

Sensitivity to glucocorticoids varies among individuals, among tissues from the same individual, and even within the same cell depending on the phase of the cell cycle (223). Hereditary studies show that differences in the glucocorticoid receptor gene make 6.6% of the normal population relatively hypersensitive to glucocorticoids, and 2.3% relatively resistant (169). Yet, glucocorticoid resistance may also be acquired and localized to the sites of inflammation (169) with pathological conditions (224). Glucocorticoid sensitivity is largely determined by a number of factors including the intracellular density and distribution of glucocorticoid receptors (183), 11βHSD1**-**mediated intracellular synthesis of active cortisol from inactive cortisone (179), tissue-specific presence of coregulatory proteins, the phosphorylation status of GR, the sequence of the GR-binding site and flanking DNA on target genes (184, 225), post-translational modifications of GR, the availability of specific co-activators and co-repressors, epigenetic regulators, the chromatin landscape (187, 190), and cross-talk with MyoD family inhibitor domain-containing proteins (226).

With exercise training, the body adapts to regulate glucocorticoid sensitivity in some cell types (172). Increased tissue sensitivity to glucocorticoids following (6–24 h) acute exercise may serve to counteract muscle inflammatory reaction and cytokine synthesis and then decrease exercise-induced muscle damage or inflammatory response (172). Subsequent decreased sensitivity of monocytes to glucocorticoids 24 h following exercise may act to protect the body from prolonged, exercise-induced cortisol secretion (172). Intracellular adaptation of glucocorticoid regulators to exercise is tissue specific, resulting in decreases in glucocorticoid action in skeletal muscle and increases in glucocorticoid action in the liver and visceral fat (227). While exercise attenuates glucocorticoid induced muscle atrophy (228), glucocorticoid exposure (via prednisolone exposure) reduces exercise performance, increases blood glucose concentrations and white blood cell counts and alters Leydig cell function (229).

## KLF15—A Target of Glucocorticoid Receptor in Skeletal Muscle

A peripheral clock system is present in a human adrenocortical cells where periodic oscillations of clock genes are influenced by glucocorticoids, mainly through GRα (230). In human leukocytes, glucocorticoid receptor expression parallels that of plasma cortisol with values peaking in the morning at 04:00–08:00 h and being lowest at 23:00–24:00 h (231). The diurnal variations in the glucocorticoid receptor may serve to coordinate the reactivity of the target cells to cortisol (231). Corresponding to the peripheral clock system are responses to glucocorticoid exposure where, although chronic and sustained exposure to glucocorticoids promotes catabolic consequences for skeletal muscle, intermittent exposure appears to have a more favorable impact (232, 233). In fact, intermittent administration of glucocorticoids appears to promote sarcolemmal repair and muscle recovery from injury (232) and muscle performance (233). In contrast, sustained glucocorticoid exposure induces muscle atrophy. Differences in muscle responses to intermittent compared to sustained exposure to glucocorticoids are likely mediated by transcription factor KLF15, which also increases with weekly exposure, but is suppressed with daily exposure (232).

Transcription factor Kruppel-like factor 15 (KLF15) is a direct target of the glucocorticoid receptor in skeletal muscle (212). Within skeletal muscle it regulates lipid utilization (234), coordinates the transcriptional circuitry responsible for metabolism (234), mediates the metabolic ergogenic effects of glucocorticoids via metabolic programming (233), and affects exercise capacity (212, 234). In addition to its metabolic role, KLF15 regulates myofiber typing (235), mTOR activity (233), and myofiber size (212). KLF15 displays a diurnal pattern of expression, and regulates branched-chain amino acid (BCAA) metabolism and utilization in a circadian fashion (236). Glucocorticoid exposure (237), acute endurance exercise (234), and hyperglycemia lead to increased KLF15 expression. As a direct target gene of the glucocorticoid receptor with a diurnal response pattern, KLF15 signaling may explain the complex role of glucocorticoids in metabolism and protein balance and mechanistically favor the intermittent value of glucocorticoids via exercise or pharmaceuticals.

## Conclusions

Hormones are largely responsible for the integrated communication network responsible for modulating cellular signaling for protein synthesis (165). All aspects from production, release, transportation, and tissue uptake to intracellular signaling affect the cell signaling and communication that govern basic activities of cells and coordinate all cellular actions. Among the “anabolic giants,” testosterone is the primary anabolic hormone in men. It's anabolic influence largely dictated through genomic and non-genomic signaling, satellite cell activation, interaction with other anabolic signaling pathways, upregulation or downregulation of the androgen receptor, and potential roles in co-activators and transcriptional activity. Growth hormones exhibit differential influences depending on the “type” of the hormone being assayed and the magnitude of the physiological stress. The actions of IGF-I are regulated by a family of binding proteins (IGFBPs 1–6), which can either stimulate or inhibit biological action depending on binding. Circadian patterning and newly discovered variants of glucocorticoid isoforms largely dictate glucocorticoid sensitivity and catabolic, muscle sparing, or pathological influence. The downstream integrated anabolic and catabolic mechanisms of these hormones not only affect the ability of skeletal muscle to generate force, they also have implications in pharmaceutical treatments (238), aging (176), metabolic syndrome (180), insulin resistance (181), and hypertension (182). Thus, advances in our understanding of hormones that impact anabolic: catabolic processes have relevance for athletes and the general population, alike.

## Author Contributions

WK, NR, WH, BN, and MF contributed to the conception of the work, drafting the article, critical revision of the article, and final approval of the version to be published.

### Conflict of Interest

MF is an employee of and owns stock in Quest Diagnostics, which provides laboratory testing services. The remaining authors declare that the research was conducted in the absence of any commercial or financial relationships that could be construed as a potential conflict of interest.
